# Conscientiousness and fruit and vegetable consumption: exploring behavioural intention as a mediator

**DOI:** 10.1080/13548506.2015.1093644

**Published:** 2015-10-22

**Authors:** Antonia E. Wilson, Daryl B. O’Connor, Rebecca Lawton, Patrick L. Hill, Brent W. Roberts

**Affiliations:** ^a^ School of Psychology, University of Leeds, Leeds, UK; ^b^ Department of Psychology, University of Illinois at Urbana-Champaign, Champaign, IL, USA

**Keywords:** Conscientiousness, behavioural intention, personality, health, fruit and vegetable consumption, Theory of Planned Behaviour, five a day

## Abstract

Clear associations have emerged between conscientiousness and health behaviours, such that higher levels of conscientiousness are predictive of beneficial health behaviours. This study investigated the conscientiousness-fruit and vegetable consumption relationship and whether behavioural intention mediated this relationship. A large sample of adults (*N* = 2136) completed an online battery of questionnaires measuring conscientiousness, behavioural intentions to consume fruit and vegetables, together with self-reported behaviour. Correlation analysis revealed that conscientiousness and each of its facets were positively associated with behavioural intention and self-reported behaviour. Hierarchical multiple regression analyses revealed that after controlling for age, gender and education, total conscientiousness, and the facets of responsibility, industriousness, order and virtue predicted self-reported behaviour. Further analysis revealed that in line with the Theory of Planned Behaviour, behavioural intention fully mediated the conscientiousness-fruit and vegetable behaviour relationship. In conclusion, low levels of conscientiousness were found to be associated with lower fruit and vegetable intentions, with the latter also associated with fruit and vegetable consumption.

## 1. Introduction

Conscientiousness is a personality trait characterised by the propensity to follow socially prescribed norms and rules regarding impulse control and to be goal directed, planful, and able to delay gratification (John & Srivastava, 1999). Over recent years clear associations have emerged between conscientiousness and longevity; with higher levels of conscientiousness predicting greater longevity (Kern & Friedman, 2008). Further research has indicated that individuals who score higher on measures of conscientiousness often engage in more beneficial health behaviours (Bogg & Roberts, 2004) and have better physical health (Moffitt et al., 2011).

More recent research, including the current study, has focused upon the mechanisms through which conscientiousness may convey such beneficial health effects. Research from Conner and Abraham (2001) found that conscientiousness was significantly associated with behavioural intentions to form health protective goals. Therefore, it seems that individuals who score high on conscientiousness may be more likely to form stronger intentions with regards to their health behaviours. Research from de Bruijn, Brug, and Van Lenthe (2009) demonstrated that individuals high in conscientiousness had a significantly higher intake of fruit than those low in conscientiousness, and that this relationship was mediated via the Theory of Planned Behaviour variables (TPB; Ajzen, 1998, 1991), as well as action planning (de Bruijn, 2013).

The majority of research exploring the relationship between conscientiousness and eating behaviour has examined unhealthy eating behaviour (Bogg & Roberts, 2004) with a couple of notable exceptions (de Bruijn et al., 2009; de Bruijn, 2013). However, few studies (if any) have adopted a facet level approach. An important study by Roberts, Chernyshenko, Stark, and Goldberg (2005) revealed that conscientiousness was best characterised by six lower-order facets: industriousness, responsibility, order, self-control, traditionalism and virtue (see also Green, O’Connor, Gartland, & Roberts, in press). Moreover, research is emerging indicating that lower order facets of conscientiousness have differential effects on health behaviours (e.g. Gartland, O’Connor, Lawton, & Ferguson, 2014; O’Connor, Conner, Jones, McMillan, & Ferguson, 2009). The facets of industriousness and traditionalism have been highlighted as being particularly important for eating behaviour (Bogg & Roberts, 2004), therefore, the current study aimed to explore the role of the lower order facets in the context of consumption of fruit *and* vegetables in a large representative sample.

In sum, we predicted that: (1) conscientiousness and its facets (in particular, industriousness & traditionalism) will be positively correlated with behavioural intentions to consume fruit and vegetables and self-reported fruit and vegetable behaviour, and (2) the effects of conscientiousness and its facets on self-reported fruit and vegetable behaviour will be mediated by behavioural intention.

## 2. Methods

### 2.1 Participants

A sample of 2136 participants were recruited across the US for a large cross-sectional study (1092 women, 1044 men)^1^ with a mean age of 50.96 years (range = 20–101 years old). Participants were largely of a Caucasian ethnicity (*N* = 1691, 79.2% of the sample) and completed the study online. The majority of participants were employed (53.5%) or retired (28.2%). Participants were recruited via the Knowledge Networks, Inc. survey administration service. This study received ethical approval from the University of Illinois’ Institutional Review Board and participants were compensated $30.

### 2.2 Measures

#### 2.2.1 Conscientiousness

Conscientiousness was assessed using the 60 item Chernyshenko Conscientiousness Scale (Green et al., in press)^2^. The facets measured were industriousness, order, traditionalism, self-control, responsibility and virtue. Each facet has demonstrated differential predictive validity (Hill & Roberts, 2011). Items were scored on a four point Likert scale with responses from disagree strongly (1) to agree strongly (4). A high score indicated a high level of conscientiousness. Scores on the six facets were averaged to create an overall score of conscientiousness (Cronbach’s *α* = .82).

#### 2.2.2 Behavioural intention

Intention to consume fruit or vegetables was assessed through the item ‘I intend to eat five fruits and/or vegetables a day’. Responses ranged from strongly agree (7) to strongly disagree (1), following the procedures outlined by Conner and Norman (2005).

#### 2.2.3 Self-reported fruit and vegetable consumption

Eating behaviour was assessed via the Behavioural Risk Factor Surveillance System (National Center for Chronic Disease Prevention and Health Promotion, 2000). Five items asked participants to report responses on a 7 point scale with responses varying from ‘I did not have any during the past 7 days’ through to ‘four or more times per day’. Items included ‘How many times did you drink 100% fruit juices such as orange juice, apple juice or grape juice?’, ‘How many times did you eat green salad?’, ‘How many times did you eat carrots?’, ‘How many times did you eat vegetables other than green salad or carrots?’ and ‘How many times did you eat fruit? (Do not count fruit juice)’. Responses to these items were averaged to create an overall score of fruit and vegetable eating behaviour, with a high score indicating a greater number of fruits and vegetables consumed (Cronbach’s *α* = .78).

## 3. Results

### 3.1 Descriptive statistics

Descriptive statistics for each measure alongside correlation coefficients between each study variable are presented in Table 1.

**Table 1.** Means, standard deviations, Cronbach’s alpha and Pearson product-moment correlation coefficients for conscientiousness, behavioural intention and self-reported behaviour (*N* = 2031–2132).

		1	2	3	4	5	6	7	8	9
1.	Conscientiousness	__								
2.	Industriousness	.79	__							
		[.77, .80]								
3.	Order	.64	.44	__						
		[.62, .67]	[.40, .48]							
4.	Traditionalism	.70	.39	.31	__					
		[.68, .72]	[.35, .43]	[.26, .35]						
5.	Self-Control	.71	.47	.32	.40	__				
		[.69, .74]	[.43, .50]	[.28, .37]	[.36, .44]					
6.	Responsibility	.80	.69	.37	.42	.55	__			
		[.78, .81]	[.66, .72]	[.34, .41]	[.38, .46]	[.52, .59]				
7.	V	.73	.45	.21	.57	.45	.54	__		
		[.71, .75]	[.41, .49]	[.17, .26]	[.53, .59]	[.41, .49]	[.50, .57]			
8.	Behavioural Intention	.21	.19	.15	.11	.10	.19	.17	__	
		[.17, .25]	[.15, .23]	[.10, .19]	[.06, .15]	[.06, .15]	[.15, .23]	[.13, .21]		
9.	Self-reported Behaviour	.11	.09	.10	.05	.05	.08	.11	.46	__
		[.07, .16]	[.04, .13]	[.06, .14]	[.01, .10]	[.01, .09]	[.03, .13]	[.06, .15]	[.42, .49]	
	Mean	3.04	3.18	2.91	2.88	3.03	3.19	3.07	4.31	2.59
	SD	.35	.49	.58	.45	.43	.41	.51	1.81	.94
	Cronbach’s *α*	.82	.86	.82	.76	.78	.75	.80	__	__

Notes:

Each of the correlation coefficients were significant at the .05 level (2-tailed). Please note, there were no gender differences between fruit consumption and vegetable consumption.

### 3.2 Testing mediation effects

The preliminary correlation analysis demonstrated that there were statistically significant relationships between conscientiousness, behavioural intention and self-reported behaviour. Therefore the analysis was continued to test for mediation (for sake of brevity, see Baron and Kenny (1986) for criteria for mediation).

Multiple regression analyses were conducted to assess each component of the proposed mediation model using the Indirect SPSS Macro (Preacher & Hayes, 2008). Within the analysis, age, gender and education were entered as control variables as previous research has confirmed the effects of these variables on levels of conscientiousness (Gartland, O’Connor, & Lawton, 2012; Noftle & Robins, 2007; Vollrath, Hampson, & Júlíusson, 2012). The effects of total conscientiousness and its facets were entered into separate analyses.

### 3.3 Behavioural intention as a mediator

#### 3.3.1 Total conscientiousness

Stage one analysis demonstrated that total conscientiousness significantly predicted behavioural intention (*B* = .90, *t* (2022) = 8.11, *p* < .001). Stage two analysis demonstrated that total conscientiousness significantly predicted self-reported behaviour (*B* = .20, *t* (2022) = 3.45, *p* < .01). Stage three results indicated that the mediator, behavioural intention, significantly predicted self-reported behaviour (*B* = .24, *t* (2022) = 22.56, *p* < .001). As conditions 1–3 for mediation were met, mediation analysis was tested using the bootstrap method with bias-corrected confidence estimates (MacKinnon, Lockwood, & Williams, 2004; Preacher & Hayes, 2004). In this present study, the 95% confidence interval of the indirect effects was obtained with 5000 bootstrap samples (Preacher & Hayes, 2008). Results of the mediation analysis confirmed the mediating role of behavioural intention in the relationship between total conscientiousness and self-reported behaviour (*B* = .21, CI = .16–.28). In addition, results indicated that the direct effect of total conscientiousness on self-reported behaviour became non-significant (*B* = −.01, *t* (2022) = −.21, *p* = *ns*) when controlling for behavioural intention, thereby suggesting full mediation.

### 3.3.2 The lower order facets of conscientiousness

The same analysis procedure utilised for total conscientiousness was repeated for each of the lower order facets. Inspection of Table 2 shows that the effects of industriousness, order, responsibility and virtue on self-reported behaviour are fully mediated by behavioural intention (see step 4).

**Table 2.** Mediation analyses testing each of the lower order facets of conscientiousness (*N* = 2023–2029).

	*β* (step 1)	*β* (step 2)	*β* (step 3)	*β* (step 4)
				
Total conscientiousness	.90*	.20*	.24*	−.01
				
Industriousness	.57*	.11*	.24*	−.02
				
Order	.37*	.13*	.24*	.04
				
Responsibility	.66*	.12*	.24*	−.04
				
Virtue	.53*	.13*	.24*	.00
				
Self-control	.30*	.04	.24*	−.03
				
Traditionalism	.32*	.06	.24*	−.01

Note:

*β* = The unstandardized beta coefficient, *p < .01.

(Step 1) The IV predicts the mediator.

(Step 2) The IV predicts the DV.

(Step 3) The mediator predicts the DV.

(Step 4) The IV predicts the DV whilst controlling for the mediator.

## 4. Discussion

The results of this large scale study have provided evidence that conscientiousness and its facets are positively correlated with behavioural intention to consume fruits and vegetables and self-reported fruit and vegetable behaviour. Moreover, the findings confirm that the effects of conscientiousness on self-reported behaviour are fully mediated by behavioural intention; when conscientiousness was conceptualised in terms of a unified construct, as well as in terms of the facets of responsibility, virtue, industriousness and order. These results are notable because they support the notion that conscientiousness exerts some of its influence via self-regulatory processes that could be targeted in future behaviour change interventions.

A secondary aim of this study was to elucidate which facets of conscientiousness were most strongly associated with fruit and vegetable consumption. A meta-analysis conducted by Bogg and Roberts (2004) demonstrated that the facets industriousness and traditionalism were the most important facets in relation to eating behaviour; which is somewhat consistent with the current findings. Moreover, the differential effects of the facets support the need to continue to investigate conscientiousness at facet and global levels.

We are aware that the observed effect sizes are considered small. However, the correlations and partial correlations found in the current study are entirely consistent with most prior research linking personality traits to health behaviours (Bogg & Roberts, 2004) and to the average effect sizes found in social and personality psychology (Fraley & Marks, 2007). That is to say, the effect sizes for most social science research result in small effect sizes. Nonetheless, the correlations have indicated an interesting relationship between behavioural intention and the facets of conscientiousness, which could be particularly important in directing future research and for informing future interventions tailored to vulnerable populations.

We acknowledge that there are a number of limitations that require further comment. First, the cross-sectional nature of the research limits the conclusions that can be drawn regarding the causal direction between conscientiousness and behaviour. Second, the behavioural intention measure was only a single item and it did not include a specific time scale. Future research ought to utilise a longitudinal design incorporating improved measures of behaviour.

## Funding

This work was supported by the Economic and Social Research Council [PhD studentship award].

## Notes

1. Note the results were substantively the same for men and women, therefore, the findings for the whole sample are presented throughout.

2. The questionnaire items were not presented randomly, with the personality items administered first in the survey.
